# Taxonomic study of the genus *Aoyuanus* Ding & Chen, with descriptions of two new species (Hemiptera, Fulgoromorpha, Delphacidae)

**DOI:** 10.3897/zookeys.822.26929

**Published:** 2019-02-06

**Authors:** Zheng-Xiang Zhou, Lin Yang, Xiang-Sheng Chen

**Affiliations:** 1 Institute of Entomology, Guizhou University, Guiyang, Guizhou, 550025, China Guizhou University Guiyang China; 2 College of Agriculture, Anshun University, Anshun, 561000, China Anshun University Anshun China; 3 Guizhou Key Laboratory for Plant Pest Management of Mountainous Region, Guizhou University, Guiyang, Guizhou, 550025, China Guizhou University Guiyang China; 4 Special Key Laboratory for Development and Utilization of Insect Resources of Guizhou, Guizhou University, Guiyang, Guizhou, 550025, China Anshun University Anshun China

**Keywords:** Delphacid, Fulgoroidea, new taxa, planthopper, taxonomy

## Abstract

The delphacid planthoppers genus *Aoyuanus* Ding & Chen, 2001 is reviewed. Two new species, *A.spathulus***sp. n.** and *A.varius***sp. n.**, are described and illustrated from China to give the genus three species in total, and the generic characteristics are redefined. A short description and illustrations are also given for *A.furcatus*. A key to all known species of *Aoyuanus* based on male genitalia is provided.

## Introduction

The planthopper tribe Delphacini Leach, 1815 (Hemiptera: Fulgoromorpha: Delphacidae: Delphacinae) occurs in all ecoregions (excluding Antarctica). It is the largest clade of Delphacidae, including approximately 1639 species in 322 genera (Bourgoin 2018), and the group promises to continue to grow as new diversity is discovered (Yang 1989; Bartlett and Kunz 2015; Ren et al. 2015; Campodonico 2017; Remes Lenicov and Brentassi 2017).

The planthopper genus *Aoyuanus* was established by Ding and Chen (2001) for a single species *Aoyuanusfurcatus* Ding & Chen, 2001 from China (Chen et al. 2001). In this paper, two new species are described and illustrated from Yunnan Province, China, which were collected from weeds along a roadside. A key is given to separate all species.

## Materials and methods

Morphological terminology follows Ding (2006). Measurements of body length equal the distance between the apex of vertex and tip of tegmen. All measurements are in millimeters (mm). Dry specimens were used for the description and illustration. Color illustrations for adult habitus were obtained by KEYENCE VHX-1000. External morphology was observed under a stereoscopic microscope (Leica Mz 12.5) and characters were measured with an ocular micrometer. The genital segments of the examined specimens were macerated in 10% KOH and drawn from preparations in glycerin jelly using Olympus CX41 and Leica MZ 12.5 stereomicroscopes. Illustrations were scanned with Canon CanoScan LiDE 220 and imported into Adobe Photoshop 6.0 for labeling and plate composition.

The type specimens of the new species are deposited in the Institute of Entomology, Guizhou University, Guiyang, Guizhou Province, China (**GUGC**).

## Taxonomy

### Aoyuanus

Taxon classificationAnimaliaHemipteraDelphacidae

Ding & Chen, 2001

Aoyuanus Ding & Chen, 2001: 328; Ding, 2006: 358.

#### Type species.

*Aoyuanusfurcatus* Ding & Chen, 2001, by original designation.

#### Diagnosis.

Small sized. Frons with median carina forked at base (Figs 5, 24, 44). Pygofer asymmetrical in posterior view (Figs 12, 31, 51). Aedeagus forked at base (Figs 15, 16, 34, 35, 54, 55). Parameres simple, asymmetrical (Figs 17, 18, 36–38, 56–59).

#### Description.

*Coloration.* General color yellow to black (Figs 1, 2, 20, 21, 40, 41). Head with vertex, frons, face and antennae yellow to dark brown (Figs 4–6, 23–25, 43–45). Postclypeus paler than frontoclypeus (Figs 5, 24, 44). Pronotum and mesonotum yellow to yellowish brown (Figs 4, 23, 43). Forewings hyaline, with dark marking at apex (Figs 4, 23, 43). Legs yellow to yellowish brown (Figs 1, 2, 21, 22, 40, 41). Abdomen black, with yellow marking at lateral margins (Figs 1, 2, 21, 22, 40, 41).

*Structure.* Head including eyes narrower than pronotum (Figs 4, 23, 43). Vertex subquadrate, apically arched or truncate, keeled carinae project or not, narrower at apex than at base, submedian carinae not uniting at apex (Figs 4, 23, 43). Frons with median carina forked between eyes, longer at middle line than wide at widest part, widest at top of ocelli, lateral carinae distinctly narrowed dorsally between eyes (Figs 4, 5, 23, 24, 43, 44). Antennae cylindrical, with basal segment shorter than second, reaching postclypeus suture (Figs 5, 24, 44). Pronotum with lateral carinae almost attaining hind margin (Figs 4, 23, 43). Posttibial spur with approximately 12–20 teeth.

*Male genitalia.* Pygofer in profile distinctly wider ventrally than dorsally, asymmetrical in caudal view. Medioventral process at ventral margin of pygofer opening (Figs 10–12, 29–31, 49–51). Diaphragm broad, with dorsal margin incised, V-shaped (Figs 12, 31, 51). Aedeagus forked at base (Figs 15, 16, 34, 35, 54, 55). Parameres simple, asymmetrical (Figs 17, 18, 36–38, 56–59). Suspensorium present, Y-shaped, relative length between anal segment and aedeagus (Figs 19, 39, 60). Anal segment with a pair of processes or none (Figs 13, 14, 32, 33, 52, 53).

#### Distribution.

Oriental region (China).

#### Remarks.

This genus is extremely similar to *Indozuriel* Fennah, 1973 in appearance and is similar to *Javesella* Fennah, 1963 in shaped of aedeagus, but can be easily distinguished from the latter by the pygofer asymmetrical in caudal view (pygofer symmetrical in caudal view in *Indozuriel* and *Javesella*); parameres asymmetrical (parameres symmetrical in *Indozuriel* and *Javesella*).

##### Key to species of genus *Aoyuanus* (males)

**Table:** 

1	Pygofer with three medioventral processes (Fig. 12); anal segment without process (Figs 13, 14)	*** A. furcatus ***
–	Pygofer with a medioventral process (Figs 31, 51); anal segment with a pair of processes (Figs 32, 33, 52, 53)	**2**
2	Medioventral process not forked at apex (Fig. 31); anal segment with processes symmetrical, broad at apex (Figs 32, 33)	***A.spathulus* sp. n.**
–	Medioventral process forked at apex (Fig. 51); anal segment with two asymmetrical processes, pointed at apex (Figs 52, 53)	***A.varius* sp. n.**

### Aoyuanus furcatus

Taxon classificationAnimaliaHemipteraDelphacidae

Ding & Chen, 2001

Figs 1–9 , 10–19

Aoyuanus furcatus Ding & Chen, 2001: 328–329, figs 33–40; Ding, 2006: 358–360, fig. 189.

#### Material examined.

15 ♂♂, China: Hubei, Yingshan County, Dabieshan, 2 Jul 2014, Z-X Zhou. 6 ♂♂, Hunan, Wugang County, Yunshan, 18 Aug 1999, X-S Chen.

#### Short redescription.

Pygofer and parameres asymmetrical. Suspensorium ring-shaped, membranous.

#### Distribution.

Hubei, Guizhou, Hunan, Zhejiang.

#### Remarks.

Based on the illustrations (Figs 1–19) and description by Ding and Chen (2001), this species can be distinguished from other species of the genus by the following characters: pygofer with three medioventral processes; anal segment without process, suspensorium ring-shaped. This species is also similar to *Indozurielrostri* Ding & Zhou, 1973 in appearance, but can be easily distinguished from the latter by the parameres without curved apically (parameres with curved apically in *I.rostri*). This species is also similar to *Javesellasalina* (Haupt, 1924) in the shaped of aedeagus, but can be easily distinguished from the latter by the pygofer having a medioventral processes (pygofer without medioventral process in *J.salina*).

**Figures 1–9.**
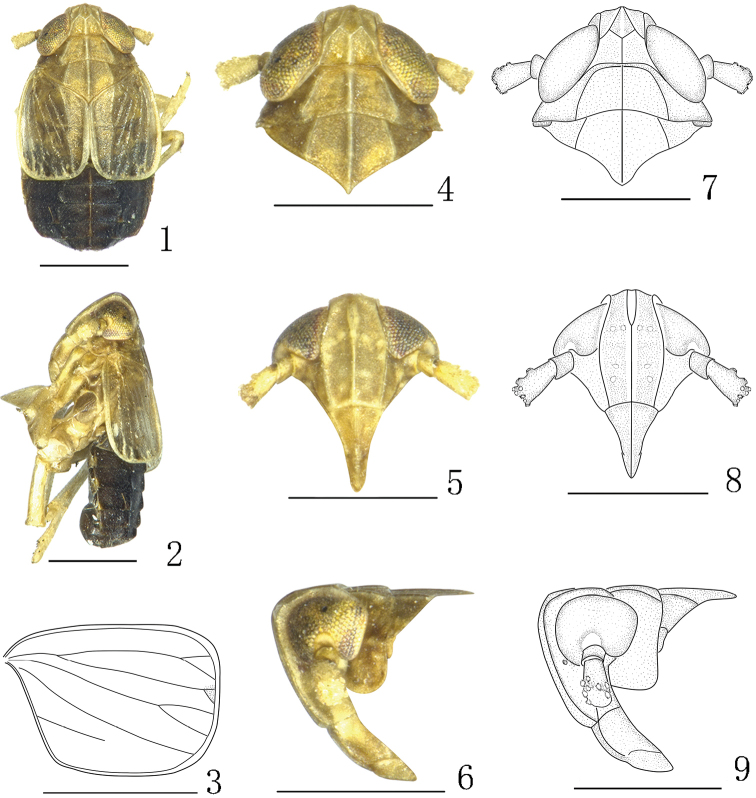
*Aoyuanusfurcatus* Ding & Chen, 2001, male. **1–2** male habitus (dorsal and lateral views) **3** forewing **4, 7** head and thorax, dorsal view **5, 8** front **6, 9** head and thorax, lateral view. Scale bars: 0.5 mm.

**Figures 10–19.**
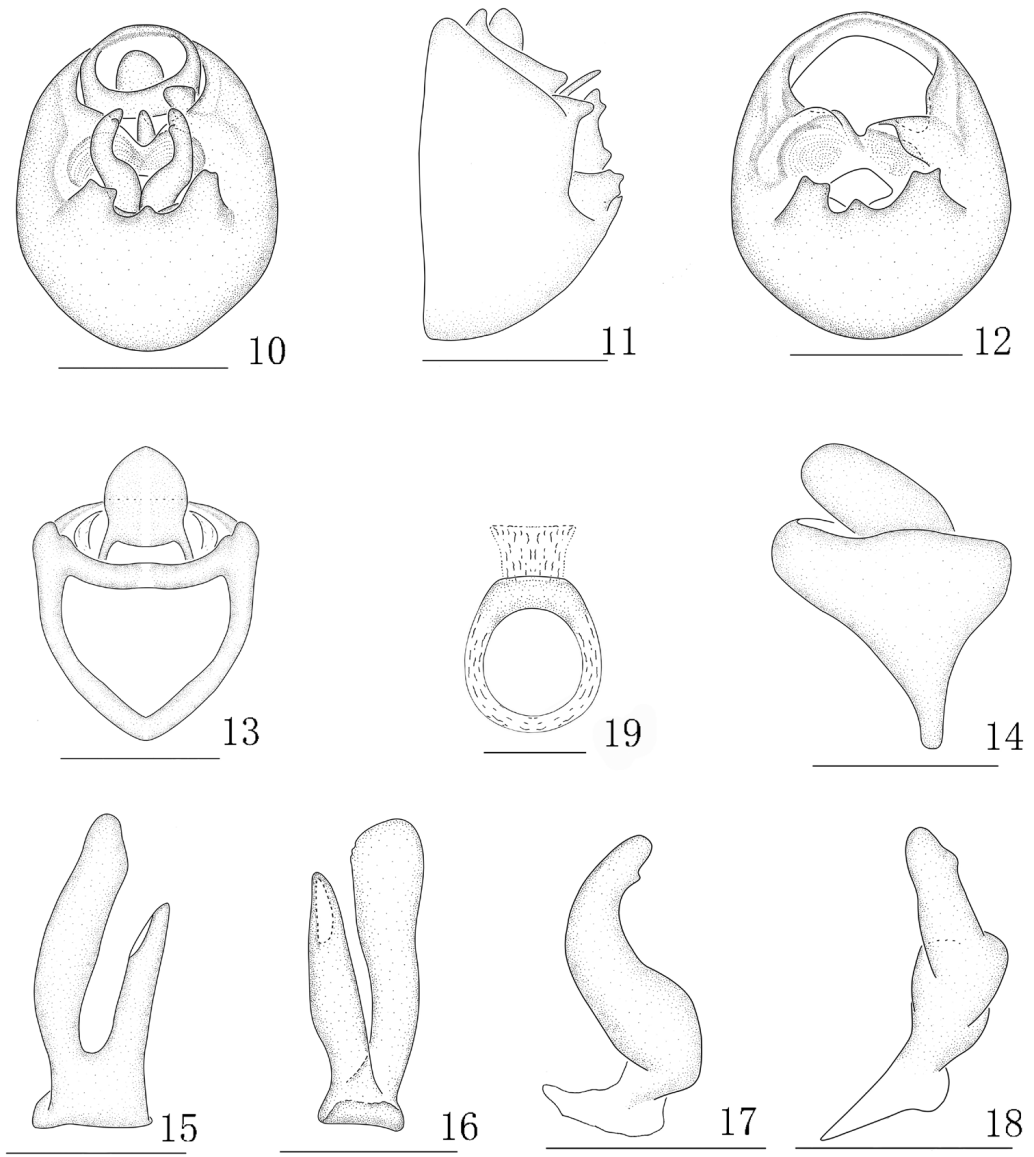
*Aoyuanusfurcatus* Ding & Chen, 2001, male. **10** genitalia, caudal view **11** genitalia, lateral view **12** diaphragm, caudal view **13** anal segment, caudal view **14** anal segment, left view **15** aedeagus, left view **16** aedeagus, ventral view **17** left paramere, caudal view **18** left paramere, left lateral view **19** suspensorium. Scale bars: 0.2 mm (**10–14**); 0.1 mm (**15–19**).

### Aoyuanus spathulus sp. n.

Taxon classificationAnimaliaHemipteraDelphacidae

http://zoobank.org/0B63176F-93FB-454A-8F80-0E271F0148B4

Figs 20–28 , 29–39

#### Type material.

Holotype: ♂, **China**: Yunnan, Simao County, Caiyanghe (22°56'N, 101°20'E), 23 Aug 2014, Z-X Zhou. Paratypes: 2♂♂, same data as holotype.

#### Type locality.

China: Yunnan, Simao County, Caiyanghe (22°56'N, 101°20'E), 1368 m.

#### Measurements

(n = 3). Body length (from apex of vertex to apex of forewing): male 1.90–2.00 mm; forewing length: male 0.80–0.87 mm.

#### Diagnosis.

**Figures 20–28.**
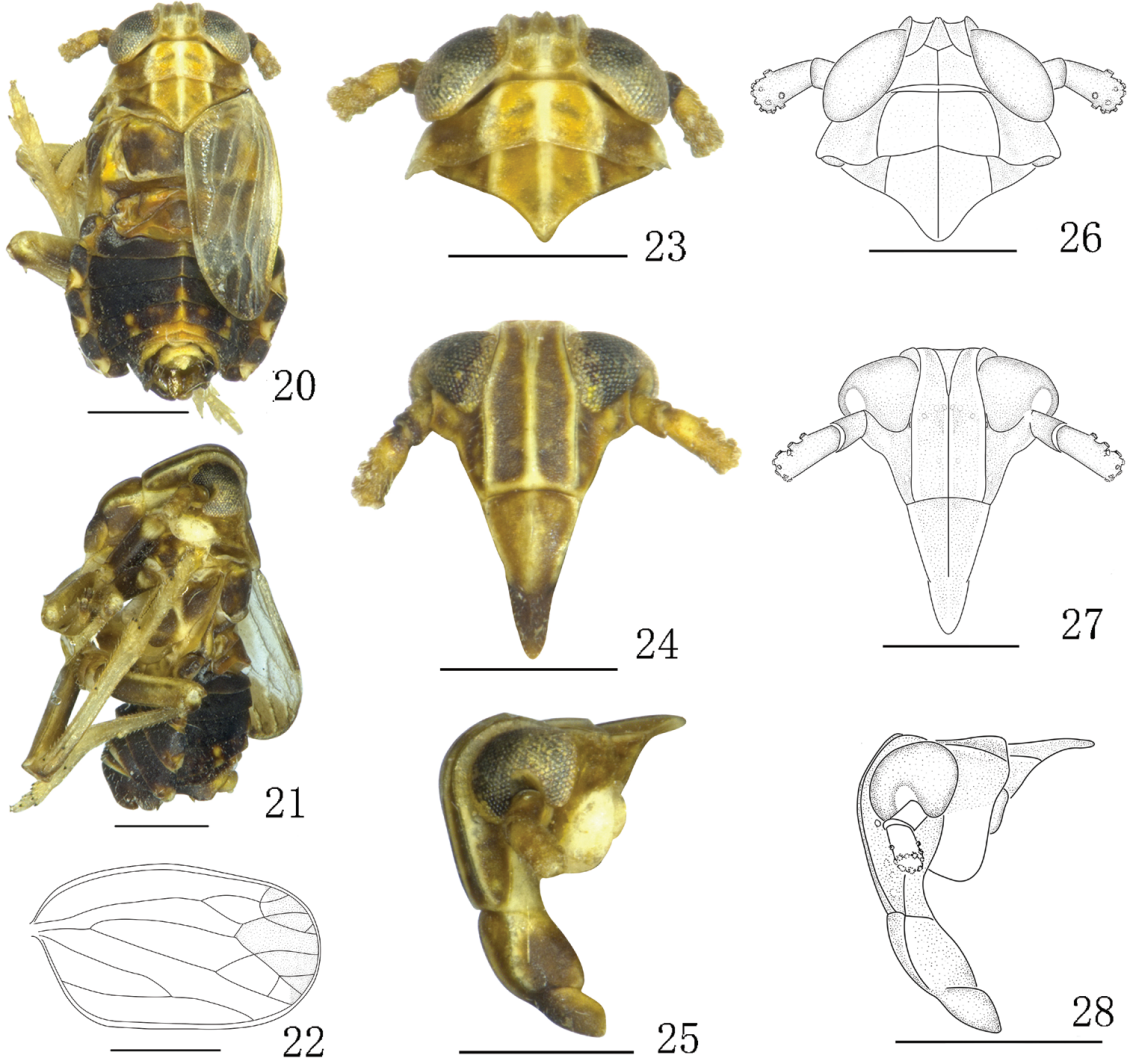
*Aoyuanusspathulus* sp. n., male. **20–21** male habitus (dorsal and lateral views) **22** forewing **23, 26** head and thorax, dorsal view **24, 27** front **25, 28** head and thorax, lateral view. Scale bars: 0.5 mm.

Forewings with brown marking at apex (Figs 20, 21). Aedeagus forked at approximately basal one-third, with dorsal one broad, flat and rounded at apex (Figs 34, 35). Anal segment with two processes at laterocaudal margins, broad and large, broadened at apex (Figs 32, 33).

**Figures 29–39.**
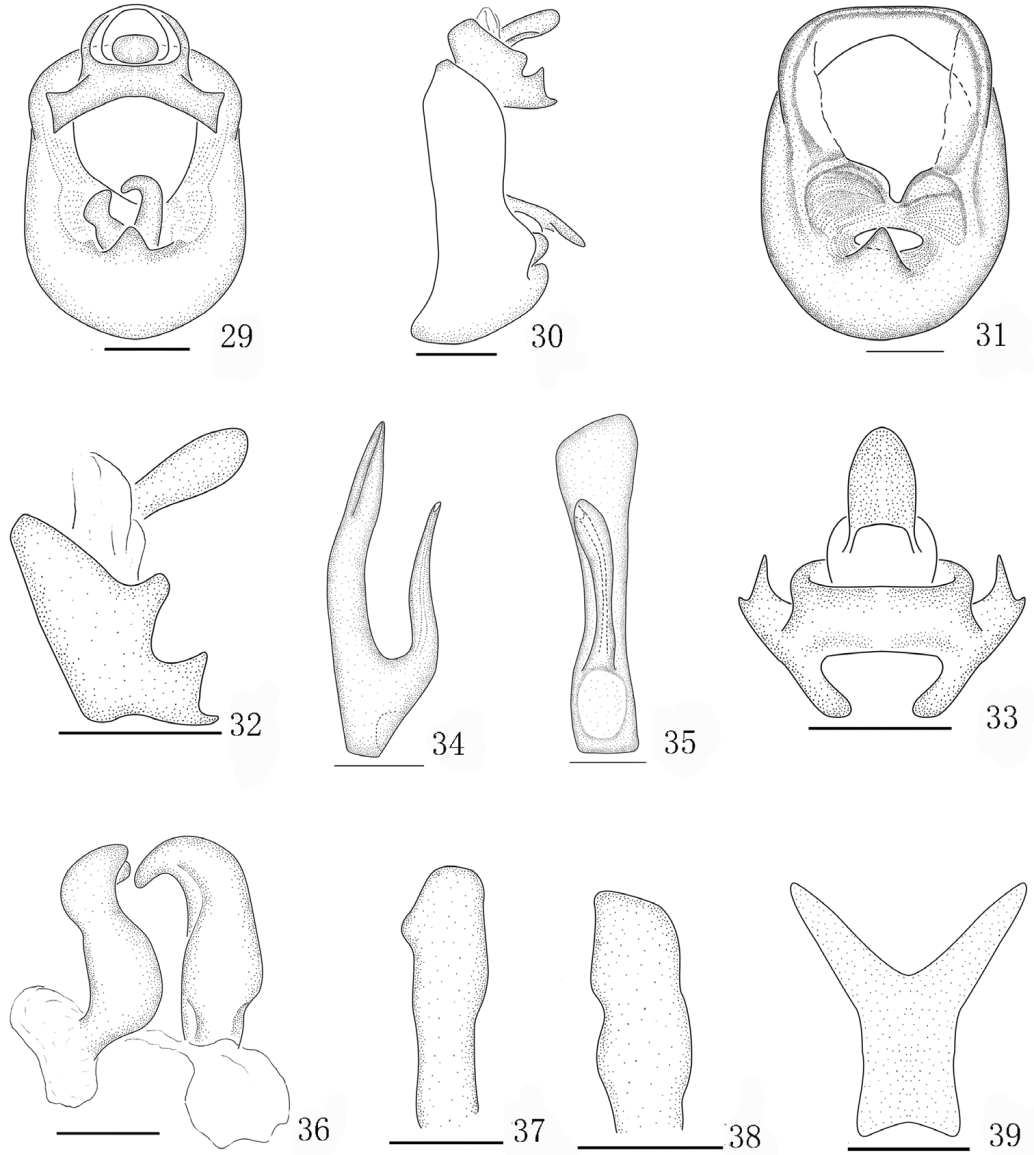
*Aoyuanusspathulus* sp. n., male. **29** genitalia, caudal view **30** genitalia, lateral view **31** diaphragm, caudal view **32** anal segment, left view **33** anal segment, caudal view **34** aedeagus, left view **35** aedeagus, ventral view **36** parameres, caudal view **37** left paramere, left lateral view **38** right paramere, right lateral view **39** suspensorium. Scale bars: 0.2 mm (**29–33**); 0.1 mm (**34–39**).

#### Description.

*Coloration.* General color yellowish white to dark brown (Figs 20, 21, 23–25). Head yellowish white to yellowish brown (Figs 23–25). Vertex yellowish white (Fig. 23). Frons black-brown, except middle carina and lateral margins yellow (Fig. 24). Clypeus yellowish brown (Fig. 24). Rostrum yellowish brown, with apex brown. Genae yellowish brown (Figs 24, 25). Eyes generally yellowish brown (Figs 20, 21, 23–25); ocellus yellow (Fig. 25). Antennae with first segment yellowish brown and black at apex and with second segment yellow (Fig. 24). Pronotum and mesonotum yellow, except carinae yellowish white (Fig. 23). Forewings with brown marking at apex (Figs 20, 21). Legs yellowish white to yellowish brown, tibiae yellow and yellowish brown basally, tarsomeres yellow (Figs 20, 21). Abdomen black, except lateral margins and middorsum with pale markings and segments 7–9 pale dorsally (Figs 20, 21).

*Structure. Head and thorax.* Head including eyes narrower than pronotum, ratio 0.98:1 (Figs 23, 26). Vertex with lateral carinae slightly concave, shorter submedially than wide at base, ratio 0.76:1, narrower at apex than at base, ratio 0.90:1 (Figs 23, 26). Frons longer in middle line than wide at widest part, ratio 1.77:1 (Figs 24, 27). Postclypeus wider at base than frons at apex, slightly longer than wide at base (Figs 24, 27). Antennae with basal segment as long as wide, shorter than second, ratio 0.45:1 (Figs 24, 27). Pronotum longer than vertex, ratio 1.06:1 (Figs 23, 26). Mesonotum shorter than pronotum and vertex combined, ratio 0.81:1 (Figs 23, 26). Posttibial spur with 16–20 distinct teeth along hind margin. Brachypterous forewings distinctly not reaching apex of abdomen, longer than widest part, ratio 1.76:1, widest at middle (Fig. 22).

*Male genitalia.* Pygofer with a medioventral process (Figs 29–31). Aedeagus forked in approximately basal one-third and dorsal one broad and flat, rounded at apex (Figs 34, 35). Parameres small, with apical half-turned mesad (Figs 36–38). Suspensorium Y-shaped, with arms as long as stem (Fig. 39). Anal segment with two processes at laterocaudal margins, large, broadened at apex (Figs 32, 33).

#### Reported hosts.

None.

#### Distribution.

China (Yunnan).

#### Etymology.

The specific name is derived from the Latin word *spathulus* (narrow and flattened), referring to the male anal segment with two spatulate processes.

#### Remarks.

The new species is similar to *Aoyuanusfurcatus* Ding & Chen, 2001 in the shape of the aedeagus, but can be easily distinguished from the latter by the male anal segment with processes (without processes in *A.furcatus*); and the pygofer with a medioventral process (with three processes in *A.furcatus*). This new species is also extremely similar to *Aoyuanusvarius* sp. n. in the shape of aedeagus, but can be easily distinguished from the latter by the anal segment with lateroapical angles symmetrical (anal segment with lateroapical angles asymmetrical in *A.varius* sp. n.); pygofer with one medioventral process not forked at apex (pygofer with medioventral process forked at apex in *A.varius* sp. n.); suspensorium Y-shaped, with arms as long as stem (suspensorium with stem longer than arms in *A.varius* sp. n.).

### Aoyuanus varius sp. n.

Taxon classificationAnimaliaHemipteraDelphacidae

http://zoobank.org/ECED68F4-41EC-40C4-8BED-E6766E23F4D2

Figs 40–48 , 49–60

#### Type material.

Holotype: ♂, **China**: Yunnan, Dali County, Xiaguan (25°58'N, 100°22'E), 4 Aug 2006, Q-Z Song. Paratype: 1♂, same data as holotype.

#### Type locality.

China: Yunnan, Dali County, Xiaguan (25°58'N, 100°22'E), 1988 m.

#### Measurements

(n = 2). Body length (from apex of vertex to apex of forewing): male 2.00–2.02 mm; forewing length: male 1.07–1.10 mm.

#### Diagnosis.

**Figures 40–48.**
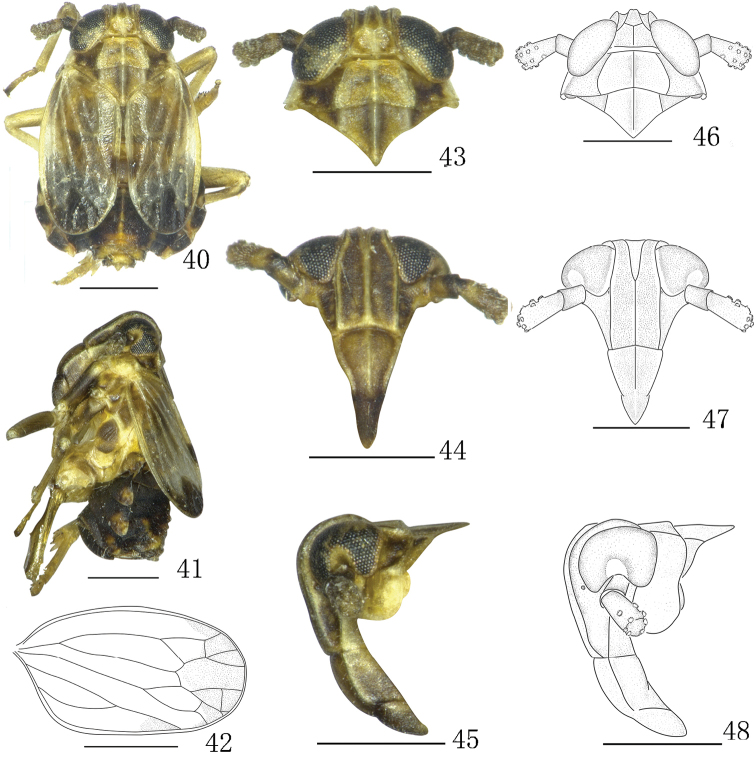
*Aoyuanusvarius* sp. n., male. **40–41** male habitus (dorsal and lateral views) **42** forewing **43, 46** head and thorax, dorsal view **44, 47** front **45, 48** head and thorax, lateral view. Scale bars: 0.5 mm.

Forewings pale yellow with dark black marking at apex (Figs 40, 41). Aedeagus forked at basal quarter, dorsal one broadened at apex in ventral view, longer than ventral one (Figs 54, 55). Anal segment with two processes at laterocaudal margins, processes asymmetrical (Figs 52, 53).

**Figures 49–60.**
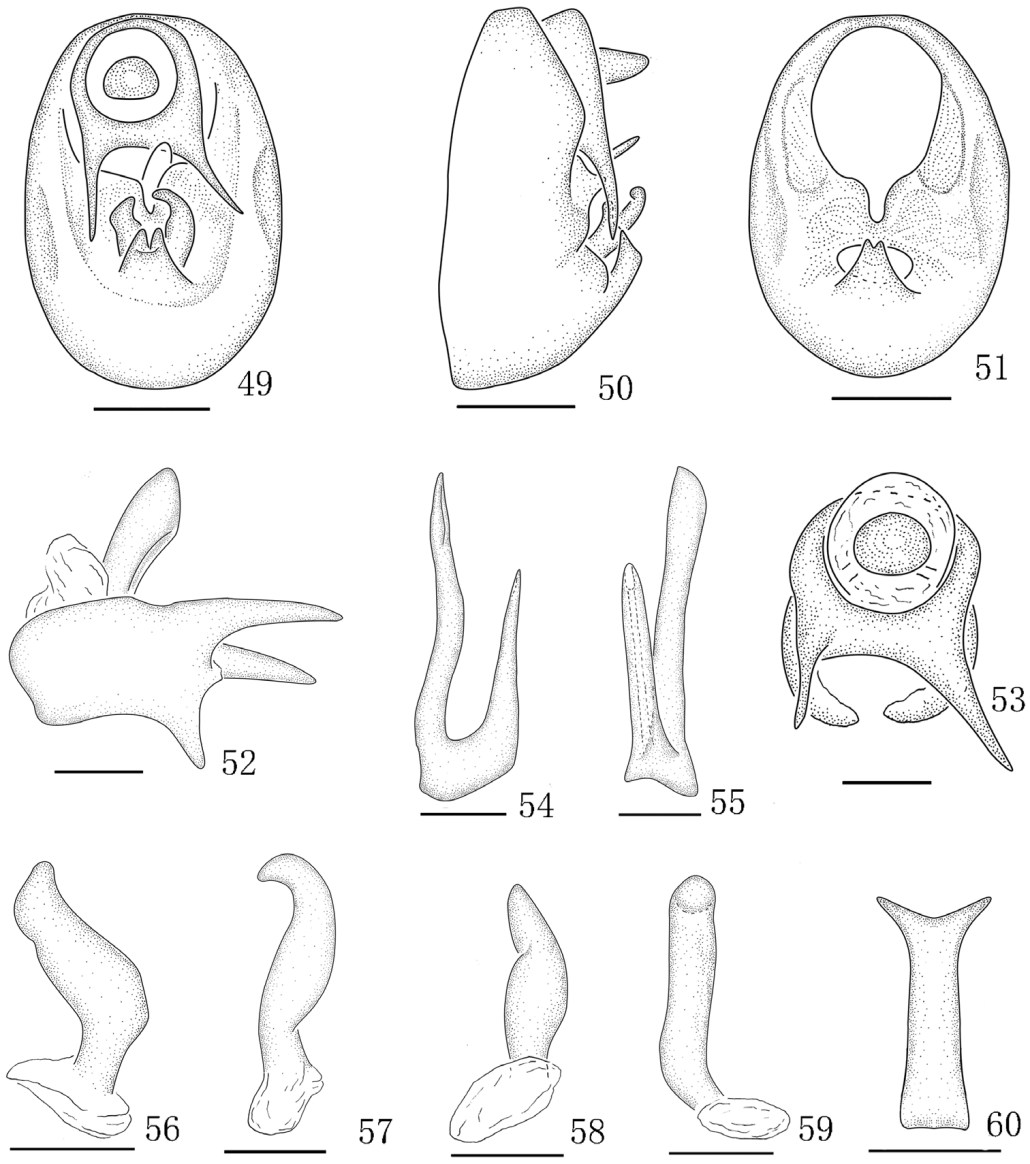
*Aoyuanusvarius* sp. n., male. **49** genitalia, caudal view **50** genitalia, lateral view **51** diaphragm, caudal view **52** anal segment, left view **53** anal segment, caudal view **54** aedeagus, left view **55** Aedeagus, ventral view **56** left paramere, caudal view **57** right paramere, caudal view **58** Left paramere, left lateral view **59** right paramere, right lateral view **60** suspensorium. Scale bars: 0.2 mm (**49–53**); 0.1 mm (**54–60**).

#### Description.

*Coloration.* General color yellow to black (Figs 40, 41, 43–45). Head yellow to yellowish brown (Figs 40, 41, 43–45). Vertex yellowish brown (Figs 40, 43). Frons black brown, except middle carina and lateral margins yellowish brown (Fig. 44). Clypeus yellowish brown to dark brown (Fig. 44). Rostrum yellowish brown, with apex brown. Genae yellowish brown (Figs 44, 45). Eyes usually yellowish brown (Figs 40, 41, 43–45); ocellus black (Fig. 45). Antennae with basal segment yellowish brown and black at apex and with second segment yellowish brown (Figs 40, 41, 43–45). Pronotum and mesonotum yellowish brown (Figs 40, 43). Forewings pale yellow with dark black marking at apex and midline dorsal margin (Figs 40, 41). Legs yellow to yellowish brown, tibiae yellow and yellowish brown basally, tarsomeres yellow (Figs 40, 41). Abdomen black, except lateral margins and dorsal apex with some small yellow markings (Figs 40, 41).

*Structure. Head and thorax.* Head including eyes narrower than pronotum, ratio 0.92:1 (Figs 43, 46). Vertex with longer than wide at base, ratio 1.18:1, narrower at apex than at base, ratio 0.81:1 (Figs 43, 46). Frons longer in middle line than wide at widest part, ratio 1.76:1 (Figs 44, 47). Postclypeus wider at base than frons at apex, slightly longer than wide at base (Figs 44, 47). Antennae with basal segment longer than wide, ratio 1.4:1, shorter than second, ratio 0.58:1 (Figs 44, 47). Pronotum shorter than vertex, ratio 0.87:1 (Figs 43, 46). Mesonotum shorter than pronotum and vertex combined, ratio 0.54:1 (Figs 43, 46). Posttibial spur with 18 distinct teeth along hind margin. Brachypterous forewings distinctly not reaching apex of abdomen, longer than widest part, ratio 1.73:1, widest at middle (Figs 40, 41).

*Male genitalia.* Pygofer with one medioventral process, which forked at apex (Figs 49–51). Aedeagus forked at basal quarter, dorsal one longer and broader at apex than ventral one in ventral view (Figs 54, 55). Parameres small, with apical half-turned mesad (Figs 56–59). Suspensorium Y-shaped, stem longer than arms, ratio 2.5:1 (Fig. 60). Anal segment with two processes at laterocaudal margins, processes asymmetrical (Figs 52, 53).

#### Reported hosts.

None.

#### Distribution.

China (Yunnan).

#### Etymology.

The specific name is from the Latin word *varius* (meaning different, changeable), which alludes to the anal segment with two processes, which are asymmetrical.

#### Remarks.

This new species is extremely similar to *Aoyuanusspathulus* sp. n., but can be distinguished from them by the anal segment with lateroapical angles asymmetrical (anal segment with lateroapical angles symmetrical in *A.spathulus* sp. n.).

## Discussion

The genus *Aoyuanus* was erected by Chen and Ding (2001) and placed in the tribe Delphacini because of its spinal formula of the hind leg 5–7–4, large, thin and flattened tibial spur, bearing a row of fine and black-tipped teeth on the posterior margin, a developed diaphragm, and the presence of a suspensorium (Yang 1989; Ding 2006). It can be distinguished from other genera of the tribe Delphacini by the pygofer and parameres that are asymmetrical in caudal view. In this study, although the suspensorium of the two new species are Y-shaped, we place them in *Aoyuanus* because of their small size; the head which includes eyes narrower than pronotum; the pygofer and parameres that are asymmetrical in caudal view; and the aedeagus forked at its basal third, with the dorsal branch flattened and ventral branch tubular. We extend the definition of the genus to include a head with the anterior margin arched or transverse, the keeled carinae may or may not project; pygofer and parameres asymmetrical in caudal view; suspensorium ring and membranous or Y-shaped.

Emeljanov (1993) established the subtribe Numatina within Delphacini, based on the region between the anal segment and phallus having a suspensorium. According to the criteria of Emeljanov (1993), *A.furcatus* Ding & Chen, 2001 belongs to the subtribe Numatina but the two new species, *A.spathulus* sp. n. and *A.varius* sp. n., do not. Nevertheless, the monophyly of the subtribe classification was not directly tested. Hence, Urban et al. (2010) considered doubtful the subtribe classification and divided Delphacini into three major clades. Recently, Huang et al. (2017) supported the general concept of subtribe Numatina and supported the division of this tribe into three clades, but they are composed differently from those of Urban et al. (2010). According to Huang et al. (2017), *A.furcatus* belongs to the subtribe Numatina, but two new species are not clearly placed. Therefore, in this paper, we provisionally place the two new species in the genus *Aoyuanus*, but more taxon samples and molecular data are still required to confirm the relationships within Delphacini in the future.

## Supplementary Material

XML Treatment for Aoyuanus

XML Treatment for Aoyuanus furcatus

XML Treatment for Aoyuanus spathulus

XML Treatment for Aoyuanus varius
